# Nutrients and Natural Substances for Hypoglycemic Effects and Management in Diabetic Retinopathy

**DOI:** 10.3390/nu17071207

**Published:** 2025-03-30

**Authors:** Francesco Cappellani, Roberta Foti, Giulia Malaguarnera, Fabiana D’Esposito, Carlo Musumeci, Lorenzo Rapisarda, Daniele Tognetto, Caterina Gagliano, Marco Zeppieri

**Affiliations:** 1 Department of Ophthalmology, University of Catania, 95123 Catania, Italy; francescocappellani@hotmail.com (F.C.);; 2 Division of Rheumatology, A.O.U. “Policlinico San Marco”, 95123 Catania, Italy; 3 Department of Human Sciences and Quality of Life Promotion, San Raffaele Roma Open University, 00166 Rome, Italy; 4 Imperial College Ophthalmic Research Group (ICORG) Unit, Imperial College, 153-173 Marylebone Rd., London NW1 5QH, UK; 5 Department of Neurosciences, Reproductive Sciences and Dentistry, University of Naples Federico II, Via Pansini 5, 80131 Napoli, Italy; 6 Department of Medicine and Surgery, University of Enna “Kore”, Piazza dell’Università, 94100 Enna, Italy; 7 Department of Medicine, Surgery and Health Sciences, University of Trieste, 34127 Trieste, Italy; 8 Mediterranean Foundation “G.B. Morgagni”, 95125 Catania, Italy; 9 Department of Ophthalmology, University Hospital of Udine, 33100 Udine, Italy

**Keywords:** diabetic retinopathy, hypoglycemia, nutrition, natural compounds, glycemic regulation, antioxidants, inflammation

## Abstract

Diabetic retinopathy (DR) is a significant microvascular consequence of diabetes mellitus (DM), resulting in visual impairment and blindness. Controlling hyperglycemia is essential for avoiding and alleviating diabetic retinopathy. Nutrients and natural compounds possessing hypoglycemic characteristics present promising supplementary approaches to conventional therapies. This review assesses the influence of nutrients and natural substances on glycemic regulation and their possible effects on diabetic retinopathy. Goal: To investigate and consolidate knowledge about nutrients and natural compounds exhibiting hypoglycemic properties and their processes in the prevention and management of diabetic retinopathy. Approaches: Extensive reviews were conducted on pertinent studies from databases including PubMed, Scopus, and Web of Science. Selection criteria encompassed papers that examined natural substances, nutrients, or dietary supplements exhibiting effects on blood glucose levels and pathways associated to diabetic retinopathy. Principal findings were encapsulated according to their mechanisms, efficacy, and safety. Outcomes: Numerous foods, including omega-3 fatty acids, vitamin D, and polyphenols (e.g., curcumin, resveratrol), have hypoglycemic properties by improving insulin sensitivity and diminishing oxidative stress. Natural substances like berberine, quercetin, and flavonoids demonstrate analogous effects, influencing pathways associated with inflammation, advanced glycation end products (AGEs), and angiogenesis, which are critical factors in the evolution of diabetic retinopathy (DR). The synergistic benefits of integrating natural medicines with conventional antidiabetic medications may enhance glycemic control and reduce retinal damage. The safety profiles of these therapies are predominantly positive; nonetheless, clinical trials are still constrained in both breadth and scale. Conclusions: Nutrients and natural compounds are promising supplementary approaches for glycemic regulation and the therapy of diabetic retinopathy. Additional research, encompassing extensive clinical studies, is required to substantiate their efficacy, determine optimal dose, and verify long-term safety. The use of these natural substances into clinical practice may improve comprehensive management of diabetes and associated consequences.

## 1. Introduction

Diabetes mellitus (DM) is a chronic metabolic disorder characterized by persistent hyperglycemia due to inadequate insulin production or impaired insulin function. As the prevalence of DM continues to rise globally, its complications have become a major public health concern [1,2]. With the steady increase in the global prevalence of DM, its complications have also emerged as a global public health problem [1,2]. Among them, diabetic retinopathy (DR) is one of the most serious microvascular complications and is found in approximately one-third of diabetic patients [3]. The global prevalence of DR among diabetic patients is 22.7% and is the primary cause of legal blindness in the working-age population of the developed world [4].

DR is a progressive condition leading to damage of the retinal microvasculature with potential subsequent vision loss or blindness unless it is treated. It goes through a series of stages from non-proliferative diabetic retinopathy (NPDR), the initial stage of the disease with microaneurysms, intraretinal hemorrhages, and exudates. NPDR can progress towards the advanced stage of proliferative diabetic retinopathy (PDR) unless properly managed. PDR is a more advanced stage with retinal ischemia leading to neovascularization with a high risk for vitreous hemorrhage and retinal detachment. The transition from NPDR to PDR is brought about by chronic hyperglycemia-induced vascular damage resulting in retinal hypoxia and the increase in the expression of angiogenic factors such as vascular endothelial growth factor (VEGF) [5].

Optimal glycemic control is essential for the prevention and control of diabetic complications like DR. Early detection and control of NPDR through tight glycemic control and regular checkups with the ophthalmologist are essential for the prevention of the severe vision-threatening complications associated with PDR [6]. The primary pathological mechanisms responsible for DR are oxidative stress, inflammation, the accumulation of advanced glycation end products (AGEs), and abnormal angiogenesis [7]. The function of various nutrients and bioactive compounds in glycemic regulation and the treatment of DR has also been investigated through different studies. Various food- and medicinal plant-derived natural compounds exhibit hypoglycemic, anti-inflammatory, and antioxidant activities and could delay the progression of DR. A few examples include omega-3 fatty acids, vitamin D, polyphenols (e.g., curcumin and resveratrol), and flavonoids (e.g., quercetin and berberine). The bioactive compounds could have the potential for a complementary treatment for DR through the inhibition of various pathological mechanisms. The natural compounds are different from the conventional therapies, which target advanced-stage complications. The natural compounds could have a preventive role through the improvement of glycemic control during the initial phases of DR progression [8,9,10,11,12,13].

This review aims to consolidate current knowledge regarding the impact of nutrients and natural compounds on glycemic control and their potential role in the prevention and management of diabetic retinopathy.

## 2. Pathophysiology of Diabetic Retinopathy

The pathophysiological mechanisms underlying DR involve complex interplay of oxidative stress, inflammation, advanced glycation end product (AGE) accumulation, and dysregulated angiogenesis. These processes result in endothelial dysfunction and increased vascular permeability, which ultimately lead to retinal ischemia and neovascularization [14].

### 2.1. Hyperglycemia and Endothelial Dysfunction

Chronic hyperglycemia is the primary contributor to the onset of DR; it causes endothelial cell damage and impairs vascular homeostasis in the retina. The raised blood glucose levels result in high oxidative stress via over generation of reactive oxygen species (ROS) in retinal cells. This oxidative burden leads to mitochondrial dysfunction, DNA damage, and apoptosis of endothelial cells, thereby damaging and compromising the integrity of the blood–retinal barrier (BRB). BRB dysfunction results in greater vascular permeability causing retinal edema and hemorrhage, which is characteristic of DR within its early stages [15,16,17].

### 2.2. Oxidative Stress and Inflammation

Hyperglycemia triggers generation of reactive oxygen species (ROS), which activate intracellular signaling pathways, such as nuclear factor-kappa B (NF-κB), and increase levels of pro-inflammatory cytokines including tumor necrosis factor-alpha (TNF-α), interleukin-1 beta (IL-1β), and interleukin-6 (IL-6). These inflammatory mediators promote vascular dysfunction by promoting leukocyte adhesion, microvascular occlusion, and capillary dropout, worsening these events and thus worsening retinal ischemia. In fact, leukocyte adhesion and leukocyte β2-integrin (CD11a, CD11b, and CD18) expression have been reported to be increased in diabetic rats and patients. In addition, endothelial cell adhesion molecules, including intercellular adhesion molecule-1 (ICAM-1), vascular cell adhesion molecule (VCAM)-1, and selectins (E-selectin), are upregulated in diabetic animals and patients. Chemokines, including monocyte chemotactic protein-1 (MCP-1) and macrophage inflammatory proteins MIP-1α and MIP-1β, are observed to be elevated in diabetic patients and are implicated in the pathogenesis of DR. Inflammatory cytokines like tumor necrosis factor-alpha (TNF-α) and interleukin-6 (IL-6, IL-8, IL-1β) are significantly overexpressed in diabetic individuals, and their concentration correlates with DR severity. When exposed to high glucose conditions, microglia are activated and begin the production of these inflammatory cytokines, which are very important for the propagation of an inflammatory response. Müller cells and astrocytes also contribute by producing more pro-inflammatory cytokines that further drive inflammation. In addition, pro-apoptotic molecules including cleaved caspase-3, Bax, and Fas are shown to be upregulated in retinal neurons in diabetic subjects and animal models. Persistent inflammation leads to further damage, worsening the progression of DR from non-proliferative to proliferative stages [18,19,20,21].

### 2.3. Advanced Glycation End Products (AGEs) and Vascular Dysfunction

The formation and accumulation of AGEs contribute significantly to the pathophysiology of DR. AGEs result from non-enzymatic glycation of proteins and lipids due to prolonged hyperglycemia. These compounds interact with their receptors (RAGE) on retinal endothelial cells, triggering oxidative stress, inflammation, and apoptosis. The AGE-RAGE interaction activates multiple downstream signaling pathways, including protein kinase C (PKC) and NF-κB, which further amplify vascular damage. AGEs also activate receptors that induce pro-oxidant and pro-inflammatory cascades, exacerbating oxidative stress and leukocyte adhesion. AGEs also disrupt pericyte function, leading to capillary degeneration and microaneurysm formation, hallmark features of early DR [22,23,24,25,26].

### 2.4. Angiogenesis and Neovascularization

As DR progresses, chronic retinal hypoxia triggers compensatory neovascularization, a hallmark of proliferative diabetic retinopathy (PDR). Hypoxia-inducible factor-1 (HIF-1) is upregulated in response to ischemia, leading to increased expression of VEGF. VEGF promotes endothelial cell proliferation and migration, resulting in the formation of fragile and leaky new blood vessels. These abnormal vessels growth increase the risk of vitreous hemorrhage and tractional retinal detachment, and these complications may result in severe vision loss in advanced DR. Anti-VEGF therapies have been developed to target this pathological angiogenesis and are currently a cornerstone in the treatment of PDR [27,28].

### 2.5. Neurodegeneration in Diabetic Retinopathy

In addition to vascular abnormalities, DR also involves retinal neurodegeneration. Retinal ganglion cells (RGCs) and other neuronal populations undergo apoptosis due to hyperglycemia-induced oxidative stress and inflammation. Dysfunction of glial cells, especially Müller cells and microglia, leads to disruption of the retinal microenvironment, which aggravates neurodegeneration in themselves [29,30,31]. Emerging evidence suggests that early neurodegenerative changes may occur before vascular pathology [32].

**Figure 1:**
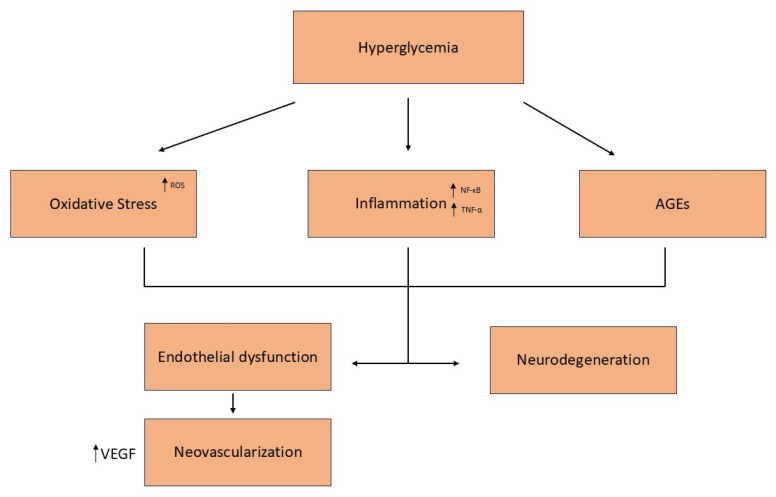
Pathophysiology of diabetic retinopathy. Key mechanisms include oxidative stress, inflammation, AGE accumulation, and VEGF-driven neovascularization, leading to vascular dysfunction, neurodegeneration, and vision loss.

Figure 1 summarizes the key mechanisms involved in the pathogenesis of diabetic retinopathy.

## 3. Nutrients and Natural Compounds and Diabetic Retinopathy

Emerging evidence suggests that certain nutrients and natural compounds possess hypoglycemic, anti-inflammatory, and antioxidant properties, which may be beneficial in managing DR. These compounds target key pathological mechanisms such as oxidative stress, inflammation, and endothelial dysfunction, providing a potential adjunct to conventional therapies.

### 3.1. Omega-3 Fatty Acids

Omega-3 polyunsaturated fatty acids (n-3 PUFAs) have been extensively studied for their potential benefits in diabetic retinopathy. Studies suggest that these essential fatty acids play a crucial role in retinal health, exerting anti-inflammatory, neuroprotective, and vasoprotective effects. Among the most studied n-3 PUFAs, docosahexaenoic acid (DHA) and eicosapentaenoic acid (EPA) have been shown to exert multiple protective effects on the retina. These fatty acids regulate inflammatory pathways by suppressing pro-inflammatory cytokines. Their impact extends beyond inflammation, as they also contribute to maintaining vascular integrity by reducing endothelial dysfunction, limiting vascular permeability; additionally, omega-3 fatty acids play a role in oxidative stress regulation by enhancing the activity of antioxidant enzymes [33,34].

In contrast, omega-6 PUFAs have been linked to a higher risk of diabetic retinopathy, potentially through mechanisms involving increased expression of adhesion molecules and promotion of leukocyte adhesion in retinal endothelial cells. Recent evidence highlights the critical role of the omega-6/omega-3 polyunsaturated fatty acids ratio (PUFAR) in the development of diabetic retinopathy. A case–control study using objectively measured serum PUFA levels found that higher PUFAR was strongly associated with a lower risk of diabetic retinopathy, with individuals in the highest tertile experiencing up to a 93% reduction in odds compared to those in the lowest tertile. This suggests that maintaining a balanced PUFA ratio may offer protective effects by modulating inflammation and angiogenesis [35].

Preclinical studies have demonstrated that n-3 PUFAs can reduce retinal vascular damage and inflammation in diabetic models. Preclinical studies indicate that n-3 PUFAs, particularly DHA, can prevent retinal vascular damage by inhibiting inflammation and enhancing endothelial progenitor cell (EPC) function. Studies in diabetic animal models indicate that a DHA-rich diet prevents the formation of acellular capillaries, reduces inflammatory markers such as IL-1β and ICAM-1, and improves EPC numbers and function. The protective effects of DHA are linked to the downregulation of acid sphingomyelinase (ASM), a key enzyme involved in sphingolipid metabolism and inflammation regulation in the retina. Inhibition of ASM leads to reduced retinal vascular pathology, suggesting that n-3 PUFAs may offer a preventive strategy against diabetic retinopathy by preserving vascular integrity and suppressing pro-inflammatory pathways [36].

In a type 2 diabetes mouse model, dietary supplementation with omega-3 PUFAs was shown to significantly maintain retinal function over time, achieving levels comparable to those observed in non-diabetic controls. Electroretinography assessments demonstrated that omega-3-enriched diets helped prevent the progressive deterioration of visual responses, while mice receiving omega-6-rich diets exhibited a marked decline in retinal function [37].

Experimental findings showed that mice fed a flaxseed oil-supplemented diet exhibited significant reductions in retinal inflammation, alongside improved electroretinographic responses. Notably, G-protein-coupled receptors 120 and 40 (GPR120 and GPR40), which mediate omega-3 fatty acid signaling, were detected in the retina. These receptors were found to be modulated by dietary omega-3 intake, further supporting the role of unsaturated fatty acids in retinal homeostasis [38].

However, translating these findings into clinical practice has been challenging, and clinical studies have yielded conflicting results. The ASCEND-Eye study, a large-scale randomized controlled trial involving 15,480 adults with diabetes, investigated the effects of daily supplementation with 1 g of omega-3 fatty acids (460 mg eicosapentaenoic acid and 380 mg docosahexaenoic acid) over an average follow-up of 6.5 years. The study found no significant difference in the incidence of referable diabetic retinopathy between the omega-3 and placebo groups [39].

The PREDIMED study indicated that higher dietary intake of long-chain omega-3 fatty acids was associated with a reduced risk of sight-threatening DR. Participants consuming at least 500 mg/day of omega-3 fatty acids had a 48% relative risk reduction in vision-threatening DR compared to those with lower intake [40].

A combined analysis of two multi-ethnic cohorts, including individuals with type 2 diabetes, found that higher levels of DHA were significantly associated with a lower likelihood of developing diabetic retinopathy. Participants in the highest quartile of DHA levels exhibited a 17% reduced risk of retinopathy compared to those in the lowest quartile. Moreover, individuals with the highest DHA concentrations also demonstrated a 38% lower severity of retinopathy, suggesting that DHA may not only reduce the risk of DR but also slow its progression [12].

In addition to these findings, recent clinical research has explored the relationship between circulating omega-3 biomarkers and retinal health in individuals with type 1 diabetes (T1D). Evidence suggests that patients with T1D, regardless of their DR stage, tend to have significantly lower blood levels of marine-derived omega-3 fatty acids—including EPA, docosapentaenoic acid (DPA), and DHA—compared to non-diabetic individuals, and higher levels of these omega-3 fatty acids have been associated with a lower prevalence of DR, while increased proportions of DPA, DHA, and EPA + DHA have been linked to higher vessel and perfusion densities in the macula [41].

Although multiple preclinical and clinical studies have explored the role of omega-3 fatty acids in DR, findings from human trials remain inconclusive. Differences in study design, dosage, baseline DR status, and population characteristics may partly explain the conflicting results. For instance, while the PREDIMED study reported a protective association with dietary intake, the ASCEND-Eye trial using supplement-based intervention found no significant effect. Moreover, variability in the form and source of omega-3s (dietary vs. pharmaceutical), adherence rates, and the lack of biomarker stratification in many trials complicate interpretation. These data have highlighted the necessity for extensive, prolonged randomized controlled trials to confirm the effectiveness of omega-3 fatty acids to evaluate optimal dosing, duration, and patient subgroups most likely to benefit in the management of diabetic retinopathy.

### 3.2. Vitamin D

Vitamin D, a fat-soluble secosteroid, plays a crucial role in bone metabolism, immune function, and vascular homeostasis. Increasing evidence suggests that vitamin D deficiency is associated with a higher risk of diabetic complications, including DR. The protective role of vitamin D in DR may be attributed to its anti-inflammatory, antioxidant, and anti-angiogenic properties, as well as its role in maintaining endothelial function. Vitamin D exerts protective effects by modulating inflammatory responses, reducing oxidative stress, preserving the blood–retinal barrier, and inhibiting pathological neovascularization [42]. Vitamin D has been shown to modulate inflammatory responses by inhibiting the nuclear factor-kappa B (NF-κB) pathway, which reduces the expression of pro-inflammatory cytokines such as TNF-α, IL-1β, and IL-6 [43].

In a diabetic rat model, vitamin D3 supplementation for six months significantly reduced retinal oxidative stress by downregulating intracellular ROS levels and inhibiting the TXNIP/NLRP3 inflammasome pathway, a key driver of inflammation in diabetic retinopathy. Treated rats exhibited lower retinal vascular permeability, decreased VEGF expression, and reduced endothelial cell apoptosis, resulting in improved retinal structure and function compared to untreated diabetic controls [44].

Moreover, calcitriol, the active form of vitamin D, reduces VEGF-induced proliferation and migration of retinal endothelial cells [45]. Animal models treated with vitamin D analogs showed a reduction in retinal neovascularization and decreased VEGF expression [46].

In a high-glucose-induced retinal pigment epithelial (RPE) cell model, vitamin D supplementation enhanced cell viability, reduced ROS production, and decreased caspase-3/7 activity, which are markers of oxidative stress-induced apoptosis. Similarly, in an STZ-induced diabetic mouse model, vitamin D-treated mice exhibited lower levels of pro-inflammatory cytokines and increased antioxidant enzyme expression [47].

Additionally, vitamin D has been shown to reduce the accumulation of AGEs, which contribute to oxidative stress-mediated vascular damage in diabetes. In a diabetic rat model, cholecalciferol supplementation for 10 weeks significantly lowered aortic Nε-(carboxymethyl)lysine (CML) deposition, decreased liver oxidative stress index, and improved serum total antioxidant capacity compared to untreated diabetic rats [48].

Recent evidence emphasized the significance of genetic variants in vitamin D receptors (VDRs). A meta-analysis involving over 8000 diabetic patients found significant associations between certain VDR gene variants (ApaI, BsmI, and FokI) and increased susceptibility to diabetic retinopathy across various populations. Individuals possessing certain VDR polymorphisms may have diminished reactivity to vitamin D supplementation, potentially influencing disease progression. Additional study is necessary to investigate the feasibility of tailored vitamin D therapies informed by genetic screening [49].

Multiple epidemiological studies have identified a strong correlation between vitamin D deficiency and DR. A meta-analysis of observational studies involving 9000+ diabetic patients found that individuals with low serum vitamin D levels had a 1.57 times higher risk of DR compared to those with normal levels [50]. Another meta-analysis, including 15 studies with 17,664 subjects, found that patients with serum 25(OH)D levels below 20 ng/mL had a two-fold increased risk of developing DR compared to those with sufficient vitamin D levels [51]. A study specifically examining Portuguese patients with type 1 diabetes found that lower vitamin D levels were significantly associated with a greater prevalence of DR [52].

Vitamin D has been increasingly recognized for its role in glucose metabolism and glycemic control. Clinical studies suggest that vitamin D supplementation improves insulin sensitivity and reduces fasting glucose levels in individuals with type 2 diabetes mellitus (T2DM). A randomized controlled trial demonstrated that vitamin D3 supplementation significantly reduced fasting blood glucose and HbA1c levels over six months while also lowering the required doses of oral hypoglycemic agents and insulin, suggesting an improvement in glycemic control. In this randomized controlled trial, patients with T2DM received tailored doses of cholecalciferol based on their baseline serum 25(OH)D levels. Those with severe deficiency (<20 ng/mL) received an attack dose of 50,000 IU weekly for 12 weeks, followed by a maintenance dose of 25,000 IU every two weeks. Patients with moderate deficiency (20–30 ng/mL) received 25,000 IU weekly for 12 weeks, followed by the same maintenance schedule. This dosing regimen was well tolerated and associated with significant improvements in glycemic parameters [53].

Another study found that vitamin D supplementation, particularly in combination with calcium, led to a significant reduction in HbA1c and fasting glucose levels after 12 weeks in T2DM patients, highlighting its potential as an adjunct therapy. In this randomized, controlled trial involving 150 participants, patients receiving 60,000 IU of vitamin D weekly—either alone or with 1000 mg/day of calcium—showed significant improvements in glycemic parameters. The group receiving both vitamin D and calcium experienced a greater reduction in HbA1c, suggesting a synergistic effect [54].

Further supporting these findings, a systematic review and meta-analysis of randomized controlled trials found that vitamin D supplementation was associated with modest reductions in fasting plasma glucose and insulin resistance markers in individuals at risk for diabetes [55].

### 3.3. Polyphenols

Polyphenols are bioactive compounds found in fruits, vegetables, tea, coffee, and medicinal plants, known for their antioxidant, anti-inflammatory, and anti-angiogenic properties. Recent studies suggest that polyphenols may play a protective role in DR by modulating oxidative stress, neuroinflammation, and endothelial dysfunction, making them potential adjuncts to conventional therapies. Moreover, polyphenols experience substantial biotransformation by the gut’s bacteria, which markedly affects their bioavailability and biological activity. Intestinal microbiota metabolize complex polyphenols into smaller, more bioactive metabolites, including phenolic acids and flavonoid-derived compounds, which demonstrate improved antioxidant and anti-inflammatory effects. This microbial metabolism is essential in regulating the gut–retina axis, influencing systemic inflammation and metabolic equilibrium. Polyphenols, such as quercetin and resveratrol, have been demonstrated to enhance the proliferation of beneficial bacteria, including Bifidobacterium and Lactobacillus, while suppressing pathogenic species linked to gut dysbiosis. These modifications enhance glucose metabolism and diminish systemic inflammation, potentially alleviating the advancement of diabetic retinopathy [56]. Several polyphenols, including resveratrol, curcumin, and epigallocatechin gallate (EGCG), have been investigated for their potential role in DR prevention and management.

#### 3.3.1. Resveratrol

Resveratrol, a stilbene polyphenol found in grapes and red wine, has demonstrated neuroprotective and vasoprotective properties in DR. It reduces oxidative stress and inhibits VEGF-mediated angiogenesis, a key driver of PDR [57].

##### Anti-Inflammatory Effects

Studies have shown that resveratrol significantly reduces retinal inflammation and oxidative damage by modulating paraoxonase 1 (PON1), a key regulator of microvascular complications in diabetes. In a diabetic rat model, resveratrol treatment restored insulin levels, reduced oxidative stress markers such as AGEs and LDL oxidation, and lowered inflammatory cytokines, including IL-1β, IL-6, and TNF-α. Additionally, VEGF expression and retinal vascular permeability were significantly decreased, suggesting that resveratrol mitigates retinal damage by suppressing angiogenic and inflammatory pathways [58].

##### Anti-Apoptotic and Neuroprotective Effects

Beyond its anti-inflammatory effects, resveratrol has been found to prevent apoptosis in the RPE and retinal endothelial cells under hyperglycemic conditions. In an experimental study using diabetic Agouti rats, resveratrol treatment normalized diabetes-induced alterations in MAPK signaling, restored expression of apoptosis-related proteins such as caspase-3 and Bcl-2, and prevented mitochondrial dysfunction in retinal cells [59].

##### Neurotransmitter Regulation

In addition to its vascular protective effects, resveratrol plays a crucial role in regulating glutamate toxicity in the diabetic retina. Studies in diabetic rats have shown that resveratrol prevents retinal dysfunction by preserving glutamate transporters and glutamine synthetase activity, which are critical in maintaining neurotransmitter balance and preventing excitotoxicity-induced neuronal damage [60].

##### Bioavailability and Clinical Applications

Resveratrol’s clinical application has been challenged by its low bioavailability and rapid metabolism, limiting its therapeutic efficacy. To address this issue, recent studies have explored nanostructured formulations of resveratrol as a strategy to enhance its stability, cellular uptake, and sustained activity in the retina. A novel resveratrol nanosuspension (RSV-NS) has been developed, demonstrating improved drug solubility and controlled release, allowing for greater penetration into retinal tissues. In vitro studies on human retinal endothelial cells have shown that RSV-NS significantly reduces VEGF-induced endothelial cell proliferation and migration without inducing cytotoxicity [61].

It is important to note that existing research is insufficient to determine the efficacy or safety of resveratrol supplementation in adults with type 2 diabetes mellitus, emphasizing the need for adequately powered, long-term randomized controlled trials evaluating clinically relevant outcomes to establish both efficacy and safety of resveratrol in diabetes management.

#### 3.3.2. Curcumin

Curcumin, the primary bioactive compound in turmeric (Curcuma longa), has demonstrated significant protective effects against DR through its antioxidant, anti-inflammatory, and anti-angiogenic properties. Studies suggest that curcumin exerts its effects by modulating key signaling pathways involved in oxidative stress, inflammation, and vascular dysfunction, making it a promising candidate for DR management.

##### Protection of the Blood–Retinal Barrier and Anti-Angiogenic Effects

In experimental models, curcumin has been shown to protect the integrity of the blood–retinal barrier BRB by preserving tight junction proteins and enhancing RPE function under hyperglycemic conditions. In a diabetic rat model, curcumin treatment significantly reduced BRB breakdown, preserved retinoid regeneration, and inhibited retinal neovascularization by decreasing VEGF expression.

##### Antioxidant and Anti-Inflammatory Effects

Furthermore, curcumin effectively prevented RPE apoptosis, thereby delaying the progression of DR [62].

Curcumin’s ability to mitigate oxidative stress and retinal vascular damage in DR has been linked to its suppression of the ROS/NF-κB signaling pathway. In retinal vascular endothelial cells exposed to high glucose, curcumin reduced ROS levels and prevented NF-κB activation, leading to decreased apoptosis and improved endothelial cell survival. This effect was confirmed in rat retinal vascular endothelial cells, where curcumin supplementation significantly lowered oxidative stress markers and suppressed inflammatory cytokines [63].

In another recent study using diabetic rats, curcumin administration decreased retinal levels of pro-inflammatory cytokines, including TNF-α, IL-1β, and IFN-γ, while also downregulating VEGF expression [64].

##### Glycemic Regulation

A study on photo-irradiated curcumin supplementation in streptozotocin-induced diabetic rats demonstrated its ability to significantly reduce blood glucose levels and lipid peroxidation while restoring antioxidant enzyme activity in the liver, kidney, and brain [65].

Curcumin has demonstrated significant hypoglycemic effects in both preclinical and clinical studies. It enhances glucose metabolism by improving insulin sensitivity, reducing fasting blood glucose, and lowering HbA1c levels. A randomized controlled trial found that daily curcumin intake led to a significant reduction in fasting blood glucose [66].

A systematic review and meta-analysis of randomized controlled trials showed that curcumin supplementation significantly reduced fasting glucose levels by an average of 8.85 mg/dL and HbA1c by 0.54%. The dosages of curcumin varied across studies, ranging from 80 to 2100 mg/day, with standardized curcumin doses between 46 and 1500 mg/day, and treatment durations from 4 to 12 weeks. However, the analysis also noted significant heterogeneity across studies and emphasized the need for larger, long-term trials to fully assess the safety and sustained efficacy of curcumin supplementation in diabetic populations [67].

##### Clinical Applications

Curcumin has been studied in a wide range of clinical settings with daily dosages typically ranging from 80 to 2100 mg/day, and durations ranging from 4 days to 30 months. Many studies used enhanced bioavailability formulations to improve systemic absorption, such as those combined with piperine or lipid-based carriers, which complicates direct dosage comparisons. Long-term studies (up to 30 months) and large randomized controlled trials involving hundreds of participants have consistently shown no evidence of systemic toxicity. Curcumin demonstrated an excellent safety profile, with most adverse effects being mild and gastrointestinal in nature (e.g., diarrhea, dyspepsia, flatulence, nausea). Serious adverse events were rare and typically context-specific [68].

A meta-analysis currently underway aims to provide high-quality evidence on the efficacy and safety of curcumin in DR by analyzing its impact on visual acuity, macular edema, and overall disease progression, which could offer critical insights for integrating curcumin into clinical practice [69], and a randomized, double-blind controlled trial is currently investigating the impact of curcumin–piperine supplementation on macular vascular density, oxidative stress markers, and inflammatory cytokines in non-proliferative DR patients [70].

#### 3.3.3. Flavonoids

Flavonoids are a diverse group of polyphenolic compounds widely distributed in fruits, vegetables, tea, and red wine, known for their potent antioxidant, anti-inflammatory, and vasoprotective effects. Accumulating evidence suggests that flavonoids may also play a role in glucose metabolism and insulin sensitivity, making them potential therapeutic agents for diabetes and its complications, including diabetic retinopathy.

Flavonoid-rich dietary polyphenols were found to downregulate inflammatory markers, reduce microglial activation, and alleviate retinal inflammation in diabetic mouse models [10,71,72].

Higher dietary intake of flavonols has been associated with a lower incidence of type 2 diabetes. A long-term cohort study following 2915 individuals in the Framingham Offspring cohort found that participants with higher flavonol intake had a 26% lower risk of developing diabetes, suggesting a potential protective role against hyperglycemia and insulin resistance [73].

Similarly, an analysis of the Danish Diet, Cancer, and Health Study revealed that individuals consuming higher amounts of flavonoid-rich foods had a significantly lower incidence of diabetes [74].

The mechanisms underlying the hypoglycemic effects of flavonoids involve multiple pathways, including the inhibition of intestinal glucose absorption, enhancement of insulin signaling, and reduction of oxidative stress in pancreatic β-cells. Experimental data suggest that flavonoids activate AMP-activated protein kinase (AMPK), a key regulator of glucose uptake and insulin sensitivity, and modulate the phosphoinositide 3-kinase (PI3K)/Akt pathway to enhance glucose transporter (GLUT4) translocation in muscle and adipose tissue [75].

#### 3.3.4. Berberine

Berberine is a natural alkaloid derived from many varieties of plants with multiple anti-inflammatory and antineoplastic activities.

##### Anti-Inflammatory Effects

It has also been shown to reduce inflammatory processes in diabetic retinopathy. Berberine, in fact, reduces the inflammatory processes activated by insulin through the inhibition of the Akt/mTOR/HIF-1α/VEGF axis [76]. Berberine also acts by reducing oxidative stress at the endothelial level and the apoptosis of Müller cells, the glial cells responsible for neurorepair, through the inhibition of the Nfkb axis by reducing the phosphorylation of Ikb, which is able to decrease the nuclear transcription of Nfkb [77].

##### Neuroprotective and Neurotransmitter-Regulating Effects

Retinal ischemia caused by diabetic pathology also involves a reduction in the production of some neurotransmitters including GABA. It is important to maintain the balance between this inhibitory neurotransmitter and the excitatory impulses sent by other neurotransmitters such as glutamate. In animal models, it has been seen that retinal ischemia involves an increase in the release of glutamate. This situation involves the activation of some axes (such as the Pkc-a axis and, in turn, the Bcl-2 axis) that lead to apoptosis. Berberine, through the decrease in the concentration of Bcl-2 and the increase in the production of GABA, reduces the inflammatory processes in diabetic retinopathy and therefore the apoptosis of nerve cells [78].

##### Hypoglycemic Effects

Berberine has demonstrated glucose-lowering effects comparable to metformin. When used as an adjunct therapy, it enhances the reduction of HbA1c, fasting plasma glucose, and 2-h postprandial glucose (2hPG). Additionally, berberine contributes to improvements in obesity and hyperlipidemia by lowering triglycerides (TG), total cholesterol (TC), and low-density lipoprotein (LDL) while increasing high-density lipoprotein (HDL) in individuals with metabolic disorders. Berberine also alleviates chronic inflammation by reducing serum levels of C-reactive protein (CRP), interleukin-6 (IL-6), and tumor necrosis factor-alpha (TNF-α). This suggests that berberine, when combined with hypoglycemic agents, provides synergistic benefits. Moreover, research indicates that berberine inhibits voltage-gated potassium (K^+^) channels in pancreatic β-cell membranes, thereby stimulating insulin secretion. However, this effect occurs only under hyperglycemic conditions, preventing the risk of hypoglycemia. A recent review of 46 clinical trials found that berberine is most commonly used at doses ranging from 500 mg to 1500 mg/day, typically divided into 2–3 daily doses, for durations of 4 to 24 weeks. These doses were associated with significant improvements in glycemic control and lipid metabolism, especially when combined with standard therapies such as metformin or lifestyle interventions [79]. Berberine functions as an insulinotropic agent by binding to KCNH6 potassium channels, accelerating their closure, and prolonging glucose-dependent cell membrane depolarization, ultimately increasing insulin secretion. This mechanism allows for berberine to act as a glucose-lowering agent exclusively in hyperglycemic states, minimizing the risk of hypoglycemia—a significant advantage over oral hypoglycemic agents (OHAs). Berberine effectively lowers FPG, HbA1c, and 2hPG in T2DM patients. It offers multiple therapeutic benefits, including improving insulin resistance, regulating glucose and lipid metabolism, exerting anti-inflammatory and antioxidant effects, and protecting pancreatic islet cells. Moreover, berberine’s inhibition of the KCHN6 potassium channel, hastening its closure and preventing the repolarization of pancreatic β-cell membranes, prolongs the membrane action potential, allowing for increased calcium (Ca^2+^) influx through voltage-gated calcium channels (VGCC), thereby enhancing insulin secretion. However, because pancreatic β-cell depolarization is glucose-dependent, berberine does not induce insulin release under low glucose conditions. Beyond glucose regulation, berberine also helps prevent and manage diabetic complications such as diabetic encephalopathy, nephropathy, and cardiomyopathy, as well as providing neuroprotective benefits for diabetic peripheral neuropathy. Additionally, berberine inhibits genes involved in fat synthesis, reduces preadipocyte differentiation into mature adipocytes, and limits lipid accumulation, suggesting a modest weight loss effect. Studies also show that berberine significantly decreases leptin levels in type 2 diabetes patients, making it particularly beneficial for obese individuals with diabetes. In terms of safety, berberine is generally well tolerated and no serious adverse effects were reported in the majority of clinical trials. Still, long-term safety data beyond 6 months remain limited, and further high-quality studies are warranted to confirm its long-term tolerability [80].

#### 3.3.5. Quercetin

Quercetin is a flavonoid widespread among plants with various anti-inflammatory and antioxidant actions that has been shown to be able to reduce inflammation, neoangiogenesis, and therefore the progression of diabetic retinopathy in both mice and human retinal endothelial cells. However current clinical evidence regarding safety profile of quercetin in the long term remains limited, and well-designed, long-term randomized controlled trials are needed to confirm its therapeutic efficacy and safety profile in the management of type 2 diabetes mellitus.

##### Anti-Inflammatory and Anti-Angiogenic Effects

The molecule carries out its action by—thanks to the stimulation of heme-oxygenase 1 (HO-1)—reducing the expression of the HMGB1/TLR4/NF-κB/NLRP3 inflammasome/IL-1β/IL-18 axis (and leading to a reduction in inflammatory processes). Furthermore, quercetin reduces the secretion of VEGF and increases the production of antioxidant factors such as brain-derived neurotrophic factor (BDNF). Thanks to these actions, quercetin is therefore able to reduce the pro-inflammatory and autophagy signals that are activated by hyperglycemia, to reduce the processes of neovascularization and to reduce the processes of cellular remodeling in a dose-dependent manner [81,82].

##### Gut–Retina Axis and Microbiota Effects

The gut–retina axis theory proposes that an imbalance in gut microbiota (dysbiosis) can lead to a decrease in short-chain fatty acids (SCFAs) while simultaneously increasing pro-inflammatory factors, potentially contributing to retinopathy by affecting blood circulation. Quercetin undergoes transformation by gut bacteria into low-molecular-weight phenolic compounds, such as 3,4-dihydroxybenzoic acid and 4-hydroxybenzoic acid, which are more readily absorbed by the body. Additionally, quercetin consumption raises plasma levels of isorhamnetin, a compound known for its anti-inflammatory effects. Importantly, quercetin and its metabolites have the ability to cross the blood–retina barrier. An imbalance in gut microbiota is generally associated with impaired blood glucose regulation, increasing the likelihood of retinal tissue damage. Addressing dysbiosis is therefore essential for overall health. Research has shown that increasing beneficial bacteria such as Lactobacillus and Bifidobacteria can enhance the release of angiotensin, which helps counteract capillary loss induced by hyperglycemia [83]. Furthermore, these bacteria promote glycogen synthesis, leading to reduced blood sugar levels.

##### Glycemic Control and Metabolic Regulation

Quercetin supplementation may help manage diabetes-related complications by stabilizing blood sugar levels through increased hexokinase activity and decreased glu-6-phosphatase and fructose bisphosphatase activity. Additionally, quercetin exhibits α-glucosidase inhibitory properties, which may contribute to its hypoglycemic effects [84].

#### 3.3.6. Epigallocatechin Gallate

Epigallocatechin gallate (EGCG), a catechin polyphenol found in green tea, has shown potential for mitigating DR progression through its hypoglycemic, anti-inflammatory, and antioxidant properties. Preclinical and clinical studies suggest that EGCG plays a role in regulating glucose metabolism and improving insulin sensitivity, making it a promising candidate for diabetic complications, including DR.

##### Antioxidant and β-Cell Protective Effects

EGCG has been shown to protect pancreatic islets and enhance insulin secretion. In a study using streptozotocin-induced diabetic mice, EGCG treatment improved islet viability and insulin secretion by activating the Nrf2 antioxidant pathway, reducing ROS production, and increasing heme oxygenase-1 (HO-1) expression, a key cytoprotective enzyme. Mice receiving EGCG had significantly lower blood glucose levels post-transplantation of pancreatic islets, supporting its role in preserving β-cell function and enhancing insulin action [85].

##### Autophagy and Neuroprotection

EGCG has been shown to modulate autophagy in Müller cells. In an experimental DR model, EGCG enhanced autophagic flux, promoted the formation of autophagosomes, and restored cargo degradation in Müller cells, protecting them from high glucose-induced apoptosis [86].

### 3.4. Lutein and Zeaxanthin

Lutein and zeaxanthin are xanthophyll carotenoids predominantly found in green leafy vegetables and eggs. These compounds accumulate in the retina, particularly in the macula, where they act as powerful antioxidants and blue light filters. Given the role of oxidative stress and inflammation in DR, lutein and zeaxanthin have gained attention for their potential protective effects against retinal damage in diabetes. Research has shown that diabetic patients tend to have significantly lower levels of these carotenoids compared to non-diabetic individuals [87].

A clinical study comparing diabetic patients with and without lutein and zeaxanthin supplementation suggests that these carotenoids have the potential to improve visual acuity, contrast sensitivity, and macular function in individuals with DR [88].

In an experimental study on diabetic rats, zeaxanthin supplementation prevented diabetes-induced increases in VEGF and intercellular adhesion molecule (ICAM-1), both of which play crucial roles in DR pathogenesis. Additionally, zeaxanthin reduced oxidative damage in the retina by stabilizing mitochondrial function and decreasing levels of lipid peroxidation and DNA damage [89].

Lutein and zeaxanthin contribute to retinal protection DR by targeting multiple pathological pathways. These carotenoids enhance antioxidant defenses by upregulating key enzymes such as glutathione peroxidase and superoxide dismutase, helping to counteract hyperglycemia-induced oxidative stress in retinal tissues. Additionally, they exert significant anti-inflammatory effects by suppressing NF-κB activation, which in turn reduces the expression of pro-inflammatory cytokines like TNF-α and IL-1β. Another crucial mechanism involves the modulation of VEGF [90].

In addition to retinal protection, lutein and zeaxanthin have been investigated for their potential effects on glycemic control. A clinical study assessing the impact of lutein and zeaxanthin supplementation on metabolic parameters in type 2 diabetes patients found a small but significant reduction in fasting blood glucose and HbA1c levels after 12 weeks, suggesting a possible role in glucose metabolism [91].

Table 1 provides a comprehensive overview of the key mechanisms of action and pathways targeted by these compounds.

### 3.5. Synergistic Effects of Natural Compounds with Conventional Therapies

Emerging evidence supports the use of natural compounds as adjuncts to conventional antidiabetic therapies due to their potential synergistic effects in improving metabolic control and reducing inflammation. Curcumin has shown synergistic effects when combined with metformin. In a diabetic rat model, the combination significantly improved glycemic control, reduced serum triglycerides and total cholesterol, and lowered oxidative stress more effectively than either agent alone [92]. Additional studies in diabetic rodent models show that curcumin augments metformin’s cardioprotective effects by modulating the JAK/STAT and Nrf2/HO-1 signaling pathways, further validating its role as an adjuvant agent [93]. Furthermore, combined curcumin and insulin therapy demonstrated greater neuroprotective and anti-apoptotic effects in diabetic retinal injury than insulin alone, supporting the rationale for integration of antioxidant-based interventions in early-stage diabetic complications [94]. Berberine has also been studied alongside conventional hypoglycemic agents such as metformin. A study found that berberine, when used as an adjunct to standard therapies, enhanced reductions in HbA1c, fasting plasma glucose, and postprandial glucose, while also improving lipid profiles and inflammatory markers, without increasing the risk of hypoglycemia [95]. Similarly, omega-3 fatty acids (from flax and fish oil) have demonstrated synergistic effects with the sulfonylurea glibenclamide. In diabetic rats, co-administration improved lipid metabolism by significantly lowering serum triglycerides, total cholesterol, and LDL, and increasing HDL levels [96]. The green tea polyphenol EGCG has also shown synergistic potential when combined with metformin. In a high-fat diet-induced diabetic rat model, the combination significantly reduced fasting blood glucose levels more effectively than either agent alone. Additionally, the combination lowered cortisol and hepatic 11β-hydroxysteroid dehydrogenase type 1 [97]. Flavonoids such as baicalein, quercetin, and luteolin have demonstrated synergistic inhibition of α-glucosidase when combined with acarbose, enhancing postprandial blood glucose control [98]. In a diabetic rat model, combination treatment with resveratrol and pioglitazone significantly reduced fasting blood glucose, HbA1c, insulin, and HOMA-IR levels. The combination also improved lipid profiles, restored antioxidant capacity and PPARγ levels, and lowered inflammatory markers (CRP, TNF-α, IL-6), suggesting a synergistic effect in mitigating diabetic complications [99]. Additionally, rutin, a flavonoid found in many plants, has demonstrated additive and synergistic effects when combined with multiple oral antidiabetic drugs such as metformin, glibenclamide, canagliflozin, and acarbose. Rutin significantly reduced hyperglycemia and HbA1c levels when combined with these drugs [100].

However, despite these promising synergistic effects, other studies highlight the potential for antagonistic interactions between natural compounds and conventional drugs. In vitro research has shown that green tea and its major catechin, EGCG, can inhibit key drug transporters, including multidrug and toxin extrusion proteins, and organic anion-transporting polypeptides, which are crucial for the cellular uptake and renal/hepatic clearance of drugs like metformin and atorvastatin. Specifically, EGCG significantly reduced metformin uptake by organic cation transporters and multidrug and toxin extrusion proteins, and inhibited organic anion-transporting polypeptide-mediated uptake of bromosulphophthalein and atorvastatin, raising concerns about possible pharmacokinetic interactions that could reduce drug efficacy [101]. In another study, (+)-catechin with acarbose showed antagonistic inhibition of α-glucosidase [98]. Additionally, co-administration of the citrus flavonoid naringenin with the PPARγ agonist pioglitazone was shown to attenuate pioglitazone’s hypoglycemic effects in diabetic mice. Naringenin exhibited weak partial PPARγ agonism and interfered with pioglitazone’s full agonist action, suggesting a functional antagonism despite not affecting pioglitazone pharmacokinetics [102]. These findings collectively underscore the potential of natural compounds to enhance the therapeutic effects of conventional antidiabetic drugs, offering improved glycemic control, lipid regulation, and anti-inflammatory benefits. However, they also highlight the importance of thoroughly understanding drug–nutrient interactions, as certain combinations may interfere with drug action or alter pharmacokinetics, potentially compromising therapeutic efficacy.

## 4. Conclusions

DR remains a leading cause of visual impairment and blindness among individuals with diabetes. Given its complex pathophysiology, which involves chronic hyperglycemia-induced vascular damage, oxidative stress, inflammation, and dysregulated angiogenesis, effective management of DR necessitates multifaceted therapeutic approaches. Conventional treatments such as glycemic control, anti-VEGF therapy, and laser photocoagulation have shown efficacy in mitigating disease progression. However, there is increasing recognition of the role that dietary and natural compounds may play as adjuncts to standard interventions.

This review consolidates existing evidence on the impact of various nutrients and bioactive compounds in modulating key pathological pathways involved in DR. Compounds such as omega-3 polyunsaturated fatty acids, vitamin D, and polyphenols (e.g., resveratrol, curcumin) have demonstrated significant anti-inflammatory, antioxidant, and neuroprotective effects. These compounds exert their protective effects by inhibiting pro-inflammatory cytokine production, reducing oxidative stress, preserving the integrity of the blood–retinal barrier, and mitigating pathological neovascularization. Moreover, specific flavonoids, berberine, and quercetin have shown promising effects in enhancing insulin sensitivity, reducing AGE formation, and improving retinal microcirculation, all of which are critical factors in the prevention and management of DR.

The hypoglycemic effects of these compounds, combined with their ability to modulate cellular and molecular pathways involved in DR, suggest that integrating nutritional strategies with conventional pharmacotherapy may offer a synergistic benefit.

Despite the promising findings from preclinical and clinical studies, several challenges remain. The heterogeneity of study designs, variations in dosages, bioavailability issues, and the lack of large-scale, long-term randomized controlled trials limit the ability to establish definitive clinical guidelines. While the safety profiles of these nutrients and natural compounds are generally favorable, further studies are needed to refine dosing regimens, assess potential drug interactions, and determine their long-term efficacy and safety in diabetic populations.

Future research is advised to standardize dosage protocols and to execute biomarker-driven clinical trials that will aid in the translation of preclinical findings into clinical practice. Additionally, it should prioritize well-designed clinical studies to evaluate the safety, efficacy, and mechanistic basis of the combination of these natural substances with conventional antidiabetic drugs in order to prevent unintended interactions that may affect drug absorption, metabolism, or therapeutic outcomes, and to support their evidence-based integration into standard diabetes care. Nutrients and natural compounds represent a promising adjunctive strategy for glycemic regulation and the management of diabetic retinopathy. Their incorporation into clinical practice, may enhance the prevention and treatment of this debilitating microvascular complication of diabetes. Continued research is essential to elucidate their full potential and to establish clear guidelines for their use in diabetic care.

## Figures and Tables

**Table 1:** Key mechanisms and pathways affected by nutrients and natural compounds in diabetic retinopathy.

Bioactive Compound	Mechanisms of Action	Pathways Affected in DR	References
Omega-3 (DHA, EPA)	Anti-inflammatory, vascular protection, enhances endothelial function, anti-inflammatory, reduces vascular permeability	NF-κB inhibition, VEGF modulation, ASM downregulation	[12,33,34,35,36,37,38,39,40,41]
Vitamin D	Anti-inflammatory, antioxidant, anti-angiogenic, preserves blood–retinal barrier, improves insulin sensitivity and reduces fasting glucose levels	NF-κB suppression, TXNIP/NLRP3 inflammasome inhibition, VEGF modulation	[42,43,44,45,46,47,48,49,50,51,52,53,54,55]
Resveratrol	PON1 modulation, anti-angiogenic, antioxidant, protects RPE, anti-inflammatory	PON1 pathway, MAPK signaling, VEGF suppression, glutamate balance	[57,58,59,60,61]
Curcumin	Antioxidant, anti-inflammatory, protects blood–retinal barrier, reduces VEGF, improves insulin sensitivity	ROS/NF-κB inhibition, caspase-3/Bcl-2 modulation, mitochondrial protection	[62,63,64,65,66,67,68,69,70]
Flavonoids	Antioxidant, anti-inflammatory, vasoprotective, inhibition of intestinal glucose absorption, enhancement of insulin signaling, reduction of oxidative stress in pancreatic β-cells	AMPK activation, PI3K/Akt modulation, GLUT4 translocation	[10,71,72,73,74,75]
Berberine	Anti-inflammatory, antioxidant, anti-angiogenic, glucose regulation, glucose-lowering effects	Akt/mTOR inhibition, NF-κB suppression, Bcl-2 modulation, inhibition of KCNH6 potassium channels in pancreatic β-cells	[76,77,78,79,80]
Quercetin	Anti-inflammatory, antioxidant, anti-angiogenic, glucose regulation	HMGB1/TLR4/NF-κB/NLRP3 pathway, VEGF suppression, gut microbiota modulation, increases BDNF	[81,82,83,84]
Epigallocatechin gallate (EGCG)	Antioxidant, anti-apoptotic, protects pancreatic islets, enhances insulin secretion	Nrf2 antioxidant activation, HO-1 expression, Müller cells autophagy regulation	[85,86]
Lutein and Zeaxanthin	Antioxidant, anti-inflammatory, VEGF modulation, blue light filtration	NF-κB suppression, VEGF modulation, upregulation of glutathione peroxidase and superoxide dismutase	[87,88,89,90,91]

## Abbreviations

AGEs	advanced glycation end products
AMPK	AMP-activated protein kinase
ASM	acid sphingomyelinase
BDNF	brain-derived neurotrophic factor
BRB	blood–retinal barrier
CRP	C-reactive protein
CML	carboxymethyl–lysine
DHA	docosahexaenoic acid
DM	diabetes mellitus
DR	diabetic retinopathy
DPA	docosapentaenoic acid
EPC	endothelial progenitor cell
EPA	eicosapentaenoic acid
EGCG	epigallocatechin gallate
FPG	fasting plasma glucose
GABA	gamma-aminobutyric acid
GPR120	G-protein coupled receptor 120
GPR40	G-protein coupled receptor 40
GLUT4	glucose transporter type 4
HbA1c	glycated hemoglobin
HIF-1	hypoxia-inducible factor-1
HO-1	heme oxygenase-1
ICAM-1	intercellular adhesion molecule-1
IFN-γ	interferon-gamma
IL-1β	interleukin-1 beta
IL-6	interleukin-6
IL-8	interleukin-8
IL-18	interleukin-18
IkB	inhibitor of nuclear factor kappa B
KCNH6	potassium voltage-gated channel subfamily H member 6
LDL	low-density lipoprotein
MAPK	mitogen-activated protein kinase
MCP-1	monocyte chemotactic protein-1
MIP-1α	macrophage inflammatory protein-1 alpha
MIP-1β	macrophage inflammatory protein-1 beta
mTOR	mechanistic target of rapamycin
NF-κB	nuclear factor-kappa B
NPDR	non-proliferative diabetic retinopathy
NLRP3	NOD-like receptor pyrin domain-containing 3
Nrf2	nuclear factor erythroid 2-related factor 2
OHAs	oral hypoglycemic agents
PDR	proliferative diabetic retinopathy
PI3K	phosphoinositide 3-kinase
PKC	protein kinase C
PUFAs	polyunsaturated fatty acids
RAGE	receptor for advanced glycation end products
RGCs	retinal ganglion cells
ROS	reactive oxygen species
RPE	retinal pigment epithelium
RSV-NS	resveratrol nanosuspension
SCFAs	short-chain fatty acids
STZ	streptozotocin
T1D	type 1 diabetes
T2DM	type 2 diabetes mellitus
TAC	total antioxidant capacity
TC	total cholesterol
TG	triglycerides
TLR4	Toll-like receptor 4
TNF-α	tumor necrosis factor-alpha
TXNIP	thioredoxin-interacting protein
VCAM-1	vascular cell adhesion molecule-1
VEGF	vascular endothelial growth factor
VDR	vitamin D receptor
VGCC	voltage-gated calcium channel
